# The New Antiseptic, "Periodate Crystals Weavers"

**Published:** 1890-03-01

**Authors:** Cecil D. Greenwood

**Affiliations:** Campbell House, Clapham Common, S.W.


### Editor's Letter-Box.

[Our Correspondents are reminded that prolixity is a great bar to publication, and that brevity of style and conciseness of statement greatly facilitate early insertion.]

### THE NEW ANTISEPTIC, "PERIODATE CRYSTALS WEAVERS."

In further illustration of the uses and efficacy of the new antiseptic, "Periodate Crystals Weavers," I send you notes of a few more cases :—

1. Scarlatina.—Called in to A. J., aged seven, on January 18th. Temp., 103 deg., tonsils much swollen, no rash. Gave Quinia Sulpt. grs. iv. at once and Tn. aconiti 7 i. every half-hour. 19th.—Temp. 104 deg., pulse 156, all symptoms worse, throat looking very bad, rash fully out. Stopped aconite, and gave "Periodate" solution (1 in 500) m.x every two hours in water. 20th.—Temp. 100$\frac{3}{5}$, pulse 112, throat much better, able to swallow much more easily same evening (*i.e.*, thirty-six hours after beginning periodate), the temperature had fallen to 99, and the rash was fading rapidly. 21st.—Patient convalescent, eating fairly well, and wanting to get up. The desquamation was comparatively slight, though sufficiently well marked for diagnosis.

2. Circumcision in a child aged six.—Usual operation dressed with a strip of lint soaked in periodate solution (1 in 500). Healed by "first intention," without the formation of a drop of pus. The rag was kept saturated with the solution.

3. Obstinate diarrhœa.—Adult, aged 28, had influenza, which was followed by diarrhœa for three weeks. After trying various remedies, including carbolic acid, without success, gave periodate, grs. v internally, and in xv of the solution every four hours. Diarrhœa ceased within 24 hours, and the patient had a healthy stool without griping the following day.

4. Severe scald of arm.—Was dressed with carron oil in the usual way. After two days there was considerable discharge of pus, and the wounds looked unhealthy. Dressed with periodate solution, the arm soon assumed a healthy appearance and the sores began to granulate.

In several other cases of minor injuries, the healing properties of the periodate solution have been well marked, and two cases of chronic gleet yielded rapidly to the same agent, especially when used internally as well as locally.

I have jotted down these few cases in the hope of inducing those who have more opportunities than myself, to give the salt a trial and to report on its merits.

Cecil D. Greenwood, M.R.C.S., L.S.A.

Campbell House, Clapham Common, S.W.

